# Is inclusion of Sabouraud dextrose agar essential for the laboratory diagnosis of fungal keratitis?

**DOI:** 10.4103/0301-4738.64122

**Published:** 2010

**Authors:** Sujata Das, Savitri Sharma, Sarita Kar, Srikant K Sahu, Bikash Samal, Aparajita Mallick

**Affiliations:** Cornea and Anterior Segment Service India; 1 Ocular Microbiology Service, L V Prasad Eye Institute, Bhubaneswar, Orissa - 751 024, India

**Keywords:** Blood agar, chocolate agar, diagnosis, fungal keratitis, Sabouraud dextrose agar

## Abstract

**Purpose:**

To determine whether the inclusion of Sabouraud dextrose agar (SDA) is essential in the diagnosis of fungal keratitis.

**Materials and Methods:**

Corneal scrapings of 141 patients with microbial keratitis were smeared and cultured. Sheep blood agar (BA), chocolate agar (CA), SDA, non-nutrient agar (NNA) with *Escherichia coli* overlay, and brain heart infusion broth (BHI) were evaluated for time taken for growth and cost. The media were also evaluated experimentally for rate of growth and time taken for identification.

**Results:**

Twenty-six of 39 patients positive for fungus in corneal scrapings by microscopy were culture-positive. Fungus grew on BA in 22/39, on CA in 18/39, on SDA in 17/39, on NNA in 17/39, and on BHI in 13/39 cases. Growth on SDA was higher in ulcers with larger infiltrate (6/18 versus 9/13, *P* = 0.04). Estimated saving with inclusion of only BA/CA was Rs. 600 per patient. Performance of all media was similar in in *vitro* experiment although the characteristic spores and color were seen earlier on SDA.

**Conclusion:**

Fungal keratitis can be reliably confirmed on BA or CA, which support growth of both bacteria and fungus.

Fungal keratitis accounts for 30-40% of all cases of microbial keratitis in developing countries.[1‐3] Treatment of microbial keratitis is aimed at rapid eradication of the infecting organisms with control of inflammation and tissue damage, thereby preserving the transparency of the cornea. Effective treatment depends on efficient identification of the infecting microorganisms.

A major factor in the improved management of fungal infection has been the ability to detect fungus, thus facilitating the selection of appropriate therapy. While some of the clinical features of fungal keratitis are suggestive of fungal infection, none of them can be considered absolutely pathognomonic of a particular type of etiological agent.[4] Therefore, microbiology workup in keratitis is mandatory before initiating any treatment.

Despite the advent of many new techniques, culture remains the cornerstone of diagnosis of most ophthalmic mycoses.[5] Sabouraud dextrose agar (SDA) has been the preferred culture medium for fungus by physicians.[6,7] While it may be ideal to inoculate onto several media,[8,9] this is not always possible due to lack of availability of media, lack of adequate sample for all media and the cost involved.

This study aimed to evaluate the usefulness of various culture media in the diagnosis of fungal keratitis, and to determine how essential is SDA in the culture of fungus. The study was done using samples (corneal scrapings) from the clinic as well as by in *vitro* experiment in the laboratory using standardized inoculum of filamentous and yeast-like fungi.

## Materials and Methods

In a pre-designed prospective study, corneal scrapings were obtained after detailed slit-lamp examination and documentation from all patients seen for non-viral microbial (bacterial, fungal, and *Acanthamoeba*) keratitis, between September 2007 and April 2008. A common protocol for diagnosis was used in all cases.

Corneal scrapings were obtained by qualified cornea specialists from the base and edge of the ulcer using a sterile surgical blade (# 15 on a Bard Parker handle) under topical anesthesia (0.5% proparacaine hydrochloride) and slit-lamp magnification. Gram, Giemsa, and potassium hydroxide with calcofluor white (KOH+CFW) stains were included as a part of the standard protocol for microscopic evaluation of corneal smears. While Gram-and Giemsa-stained smears were examined at ×400 and ×1000 magnification, the KOH+CFW preparations were examined at ×200 and ×400 magnification. Smears were examined by light-and fluorescence-microscope by a trained ocular microbiologist. Scrapings for smears were collected prior to those for culture. The presence of bacteria and fungi was reported as the number of organisms per high-power field (×400). The sequence of inoculation in the culture media was as follows: sheep blood agar (BA), chocolate agar (CA), SDA with chloramphenicol (50 μg/mL), non-nutrient agar (NNA), and brain heart infusion (BHI) broth. The samples were inoculated directly onto the solid culture media by making a row of 'C' marks. For inoculation into the liquid media, the blades were swirled directly in the culture-fluid.

SDA plates were incubated at 26±1°C to enhance the growth of fungi, and the remaining plates were incubated at 36±1°C. While CA plates were incubated with 5% carbon dioxide, BA and BHI broth were incubated aerobically, and NNA plates were incubated aerobically with an added live *Escherichia coli* suspension. All media were incubated for two weeks and were examined daily. A culture was considered significant and was reported if the smears demonstrated morphologically similar organisms, and/or if the same organism grew in more than one medium, and/or if there was confluent growth on solid media. Records were maintained on type and rate of growth, and processing of the samples for identification. The laboratory diagnoses were correlated with clinical findings.

We included all cases where the corneal scrapings were positive for fungus in direct smear examination and inoculated in all five culture media. The type, rate and time taken for growth of fungus on various culture media were analyzed. In addition, the patients were divided into two groups Group I (with infiltrate size: < 25 mm^2^) and Group II (with infiltrate size ≥ 25 mm^2^), in order to determine whether size of infiltrates had any impact on the growth of fungus.

To detect paired difference of 2±2 days between media, i.e., BA, CA, SDA, NNA and BHI, with 80% power and 0.5% significance level, a minimum sample size of 17 was required.

One each of the previously identified clinical isolates of *Aspergillus flavus* and *Candida* species were used for the experiment. Standardized inoculum was made from each by serial tenfold dilutions in normal saline to contain 10cfu/10μL for the former and 100cfu/10μL for the latter. BA, CA, BHI, SDA and NNA were inoculated in duplicates using the standardized inoculum, and they were incubated at 26±1°C for the former and at 36±1°C for the latter. The media were examined daily for number and type of colonies, and the colonies were examined for characteristic spores in lactophenol cotton blue (LPCB) mounts. NNA were examined daily under the microscope for growth and presence of characteristic features.

To analyze the cost, we looked at the annual incidence of microbial keratitis in India based on the prevalence rate of 11.3 per 10,000 population.[10] We considered the material cost of culture media and the processing cost of corneal scrapings for estimating the financial impact.

## Results

**Figure 1:**
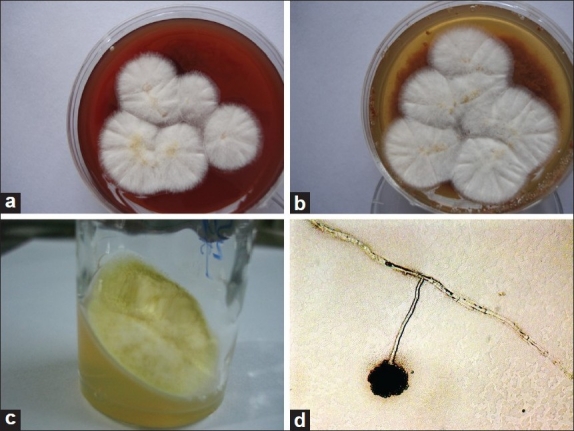
Growth of *Aspergillus flavus* on (a) Blood agar after 3-days; (b) Chocolate agar after 3-days; (c) Sabouraud dextrose agar after 4-days; (d) Non-nutrient agar after 4-days (microscopic picture, ×400)

Thirty-nine patients, with their corneal scrapings positive for fungal filaments in one or more direct smear examination, were included in the study. In 28 of 39 (72%) patients, smear was positive for fungus in all three smears (Gram, Giemsa, and KOH+CFW stain). In 26 cases (67%) the corneal scrapings were culture-positive or fungus [Fig. 1]. Of these 26 fungus culture-positive cases, eight (31%) grew fungus on all five culture media, seven (27%) grew fungus on four culture media and five (19%) grew fungus on only one culture medium. The ulcer size varied from 1 to > 64 mm^2^.

Fungus grew on BA in 22 of 39 (56%) patients, on CA in 18 of 39 (46%) patients, on SDA in 17 of 39 (44%) patients, on NNA in 17 of 39 (44%) patients and on BHI in 13 of 39 (33%) patients. In one day, the fungi grew in 11 (of 22), 10 (of 18), and 6 (of 17) cases on BA, CA and SDA respectively. Considering SDA as the preferred standard, the sensitivity of detection on BA, CA and NNA was 73%, 83% and 88% respectively; the specificity was over 80% for all [Table 1]. The kappa statistics showed variable agreement of growth on BA (k=0.50), CA (k=0.66), NNA (k=0.73) and BHI (k=0.08) to SDA. The distribution of fungal growth on various media is given in Table 2.

**Table 1:** Sensitivity, specificity and kappa coefficient of detection of fungi on BA, CA, NNA, and BHI with reference to SDA

	Sabouraud dextrose agar (SDA)		
	Sensitivity	Specificity	Kappa
Blood agar (BA)	72.7%	87.5%	0.50
	(49.5 - 88.3)	(46.6 - 99.3)	(0.20 - 0.80)
Chocolate agar (CA)	83.3%	83.3%	0.66
	(57.7 - 95.5)	(50.8 - 97.0)	(0.38 - 0.93)
Non-nutrient agar	88.2%	84.6%	0.73
(NNA)	(62.2 - 97.9)	(53.6 - 97.2)	(0.48 - 0.98)
Brain heart infusion	61.5%	47.0%	0.08
(BHI) broth	(32.2 - 84.8)	(23.8 - 71.4)	(-0.26 - 0.43)

**Table 2:** Distribution and time taken for growth of fungus on various culture media

Sl.#	BA	Day	CA	Day	SDA	Day	NNA	Day	BHI	Day
(1)	POS	1	POS	1	POS	1	POS	1	POS	2
(2)	POS	1	POS	1	POS	1	POS	1	POS	1
(3)	POS	1	POS	1	POS	1	POS	1	POS	2
(4)	POS	1	POS	1	NEG	–	NEG	–	POS	1
(5)	NEG	–	NEG	–	NEG	–	POS	2	NEG	–
(6)	POS	1	POS	1	POS	2	POS	2	POS	2
(7)	POS	1	POS	1	POS	2	POS	7	POS	2
(8)	POS	1	POS	1	POS	1	POS	1	POS	1
(9)	POS	4	NEG	–	NEG	–	NEG	–	NEG	–
(10)	POS	1	POS	1	POS	2	POS	2	NEG	–
(11)	POS	2	NEG	–	NEG	–	NEG	–	POS	1
(12)	POS	2	POS	2	NEG	–	NEG	–	NEG	–
(13)	POS	2	POS	2	POS	2	POS	2	NEG	–
(14)	POS	1	POS	1	POS	2	POS	3	NEG	–
(15)	POS	6	NEG	–	POS	3	POS	3	NEG	–
(16)	NEG	–	NEG	–	NEG	–	NEG	–	POS	12
(17)	NEG	–	NEG	–	NEG	–	NEG	–	POS	2
(18)	POS	3	POS	3	NEG	–	POS	3	POS	3
(19)	POS	2	POS	2	POS	2	POS	2	NEG	–
(20)	POS	1	POS	2	POS	1	POS	1	POS	1
(21)	POS	3	NEG	–	NEG	–	NEG	–	NEG	–
(22)	POS	2	POS	2	POS	2	POS	5	NEG	–
(23)	POS	4	NEG	–	POS	4	NEG	–	NEG	–
(24)	NEG	–	POS	5	POS	1	NEG	–	NEG	–
(25)	POS	2	POS	2	POS	2	POS	2	POS	4
(26)	POS	1	POS	1	POS	2	POS	1	NEG	–

POS: Culture-positive, NEG: Culture-negative, BA: Blood agar, CA: Chocolate agar, SDA: Sabouraud dextrose agar, NNA: Non-nutrient agar, BHI: Brain heart infusion broth

Of 26 culture-positive cases, fungus growth was observed in 50% (13 of 26), 77% (20 of 26), and 88% (23 of 26) cases within 24, 48 and 72 h respectively. Unidentified hyaline fungus and *Aspergillus spp* were the most common fungi isolated followed by *Fusarium* spp. *Aspergillus* spp was the most common when all five culture media were positive for fungus.

Growth on SDA was higher in ulcers with larger infiltrate [6 of 18 in small ulcers (< 25 mm^2^); 9 of 13 in large ulcers (≥ 25 mm^2^), *P*=0.04 (Fisher's exact test)].

The in *vitro* experiment showed the growth of *Aspergillus flavus* colonies on Day 2 on BA and SDA. But the typical yellowish green granular colonies seen on SDA on Day 2 were not seen on BA until Day 5. Spores on NNA could be seen on the fourth day with no obvious colonies. *Candida* spp grew in two days on all media except NNA. No growth was seen on NNA until the fourth day.

The estimated saving with inclusion of only BA/CA was Rs. 600 per patient.

## Discussion

While the clinical signs can only be good indicators, definitive diagnosis and thus specific therapy for microbial keratitis can only be arrived at by microbiological evaluation. It is recommended that all suspected microbial keratitis be scraped for smear and cultures before initiating antibiotic treatment.[11,12] But most community ophthalmologists do not seem to comply with the recommended practice.[13] The noncompliance might be due to lack of availability of various media, lack of adequate sample for all media and the cost involved.

Laboratory diagnosis of fungal keratitis includes direct smear examination, culture, polymerase chain reaction, and confocal microscopy. While direct smear examination provides a rapid result to clinicians for starting initial therapy, culture remains the gold standard and confirms the subjective smear results. In our institute, BA, CA, SDA, NNA, and BHI broth are routinely employed for isolation of pathogens from clinically non-viral keratitis. We deal with a large proportion of fungal keratitis patients (~30%, unpublished data) in our day-to-day work and our observation of growth of fungi on almost all media formed the basis for this study.

SDA was designed by Sabouraud for the cultivation of fungi, especially those associated with skin infections.[6] The media is more favorable for the isolation of fungi than bacteria due to its low pH and it also helps in the identification by enhancing characteristic spores and pigment production by the fungi. SDA with chloramphenicol or gentamicin (50 μg/mL) without cycloheximide is the preferred medium for corneal fungal pathogens.[14] Cycloheximide is omitted from the standard SDA used in ocular microbiology laboratories because of its ability to inhibit saprophytic fungi that are often the causative agents of fungal keratitis. Blood agar contains mammalian blood (usually sheep or horse), typically at a concentration of 5-10%. It is an enriched medium used to isolate fastidious organisms; it detects hemolytic activity. Chocolate agar is used for growing fastidious bacteria, such as *Haemophilus influenzae, Moraxella* spp, *Neisseria gonorrhoeae* and *Streptococcus pneumoniae*.

The results of this study confirm that BA and CA support the growth of all fungi commonly associated with fungal keratitis, and the time taken for growth is shorter than SDA. Therefore, exclusion of SDA from the armamentarium of culture media is unlikely to result in missing a diagnosis.

BA and CA had high culture positivity rate compared to SDA. Although SDA is the preferred standard, the growth of fungus was less in our series. This reduction in positivity may be attributed to a lower fungal load in samples due to sequential inoculation, especially in small ulcers. This leads us to expect that highest fungal load would be on BA followed by CA and SDA. A randomized study with varied sequence of plating could reveal the efficacy of various culture media in fungal keratitis.

We believe that the shorter time for growth is the result of higher load of organisms in the initial scrapings on BA and CA (first two media in the sequence of inoculation). This is corroborated by the fact that significantly higher positivity was seen in ulcers with larger infiltrates. Our in *vitro* experiment using standardized uniform inoculum on all media showed that the growth appeared simultaneously (two days) on BA and SDA for both *Aspergillus flavus* and *Candida* spp. This suggests that the time taken for growth on BA/CA and SDA inoculated with corneal scrapings from patients was indeed influenced by the size of inoculum. As expected, the identifying features of *Aspergillus flavus* were better observed on SDA. Nevertheless, since the presence of fungus is more crucial than the species identification, one may safely employ BA for management of patients with microbial keratitis of either bacterial or fungal origin. It goes without saying that an academic interest in knowing the species of fungus would make inclusion of SDA desirable. If not as a primary medium, SDA would be required to subculture the growth from BA/CA for subsequent identification.

We believe that our observation of growth of fungus on NNA (a medium traditionally used for *Acanthamoeba* culture) as observed under the microscope, is unique and has not been reported earlier. The agreement of fungal growth on NNA and SDA was excellent (k=0.73, *P* < 0.001). We routinely employ NNA with *Escherichia coli* since *Acanthamoeba* keratitis is known to mimic fungal keratitis clinically.[15,16] Fungal growth occurred on NNA in over 40% cases in this series and helped in the identification of species by virtue of *in situ* spore formation. This phenomenon was akin to a slide culture technique that is routinely employed by mycologists for identification of fungus species. In addition, NNA offered the advantage of water agar that is recommended for enhancing sporulation in fungi.[17] [Fig. 1(d)] shows the growth of *Aspergillus flavus* on the agar surface under the microscope (×400). This advantage of early identification by observation of undisturbed spore morphology without slide culture was confirmed in the in *vitro* study.

This study recommends use of blood agar or chocolate agar for diagnosis of both bacterial and fungal keratitis in situations with limited resources. Use of other media such as SDA and NNA in addition to BA/CA may be confined to clinical setups with advanced laboratory support and trained personnel.
